# Resistance-promoting effects of ependymoma treatment revealed through genomic analysis of multiple recurrences in a single patient

**DOI:** 10.1101/mcs.a002444

**Published:** 2018-04

**Authors:** Christopher A. Miller, Sonika Dahiya, Tiandao Li, Robert S. Fulton, Matthew D. Smyth, Gavin P. Dunn, Joshua B. Rubin, Elaine R. Mardis

**Affiliations:** 1 Department of Medicine, Division of Oncology, Washington University School of Medicine, St. Louis, Missouri 63110, USA;; 2 McDonnell Genome Institute, Washington University School of Medicine, St. Louis, Missouri 63110, USA;; 3 Department of Pathology and Immunology, Washington University School of Medicine, St. Louis, Missouri 63110, USA;; 4 Department of Neurological Surgery, Washington University School of Medicine, St. Louis, Missouri 63110, USA;; 5 Department of Pediatrics, Washington University School of Medicine, St. Louis, Missouri 63110, USA;; 6 Institute for Genomic Medicine, Nationwide Children's Hospital, and The Ohio State University College of Medicine, Columbus, Ohio 43205, USA

**Keywords:** ependymoma, neoplasm of the central nervous system, neoplasm of the nervous system

## Abstract

As in other brain tumors, multiple recurrences after complete resection and irradiation of supratentorial ependymoma are common and frequently result in patient death. This standard-of-care treatment was established in the pregenomic era without the ability to evaluate the effect that mutagenic therapies may exert on tumor evolution and in promoting resistance, recurrence, and death. We seized a rare opportunity to characterize treatment effects and the evolution of a single patient's ependymoma across four recurrences after different therapies. A combination of high-depth whole-genome and exome-based DNA sequencing of germline and tumor specimens, RNA sequencing of tumor specimens, and advanced computational analyses were used. Treatment with radiation and chemotherapies resulted in a substantial increase in mutational burden and diversification of the tumor subclonal architecture without eradication of the founding clone. Notable somatic alterations included a *MEN1* driver, several epigenetic modifiers, and therapy-induced mutations that impacted multiple other cancer-relevant pathways and altered the neoantigen landscape. These genomic data provided new mechanistic insights into the genesis of ependymoma and pathways of resistance. They also revealed that radiation and chemotherapy were significant forces in shaping the increased subclonal complexity of each tumor recurrence while also failing to eradicate the founding clone. This raises the question of whether standard-of-care treatments have similar consequences in other patients with ependymoma and other types of brain tumors. If so, the perspective obtained by real-time genomic characterization of a tumor may be essential for making effective patient-specific and adaptive clinical decisions.

## INTRODUCTION

Ependymomas are a heterogeneous group of primary central nervous system (CNS) tumors with multiple histological, brain region, age, and molecular features distinguishing between different prognostic groups (Pajtler et al. 2015; Dorfer et al. 2016; Khatua et al. 2017). Based on standard histological features, ependymal neoplasms can be diagnosed as World Health Organization (WHO) Grade I, II, or III tumors. However, in contrast to other brain tumors, histological grading has proven to be a weak prognostic indicator of outcome for ependymomas (Pajtler et al. 2017). In the largest published study of ependymoma outcome involving 282 patients, gross total resection (GTR) was the only prognostic factor associated with increased survival (Vera-Bolanos et al. 2015). Strikingly, in this study, GTR and postsurgical radiation therapy associated with a shorter progression-free survival than GTR alone. These data indicate that, as yet, we do not know enough about the molecular mechanisms of ependymoma, or about the appropriate indications for, and most effective modes of, adjuvant therapies.

A means to generating the necessary insights to address these concerns is comparative genomic analyses of primary and posttreatment specimens. To date, there is a paucity of information regarding the genomic changes in ependymomas that recur serially through multiple treatment regimens. This is largely due to the rarity of the disease and a failure to consistently bank and analyze recurrent samples. To determine the temporal genomic changes that occurred in one patient's ependymoma disease as it recurred after several different therapeutic modalities, we characterized the genomic landscape of serial resections with high-depth whole-genome and exome sequencing. These data provided an evaluation of putative driver mutations, mutational signatures resulting from therapy, mechanisms for therapy response and resistance, and shifts in the neoantigen profile from the initial disease presentation through four recurrences.

## CLINICAL PRESENTATION AND FAMILY HISTORY

**Figure 1.**
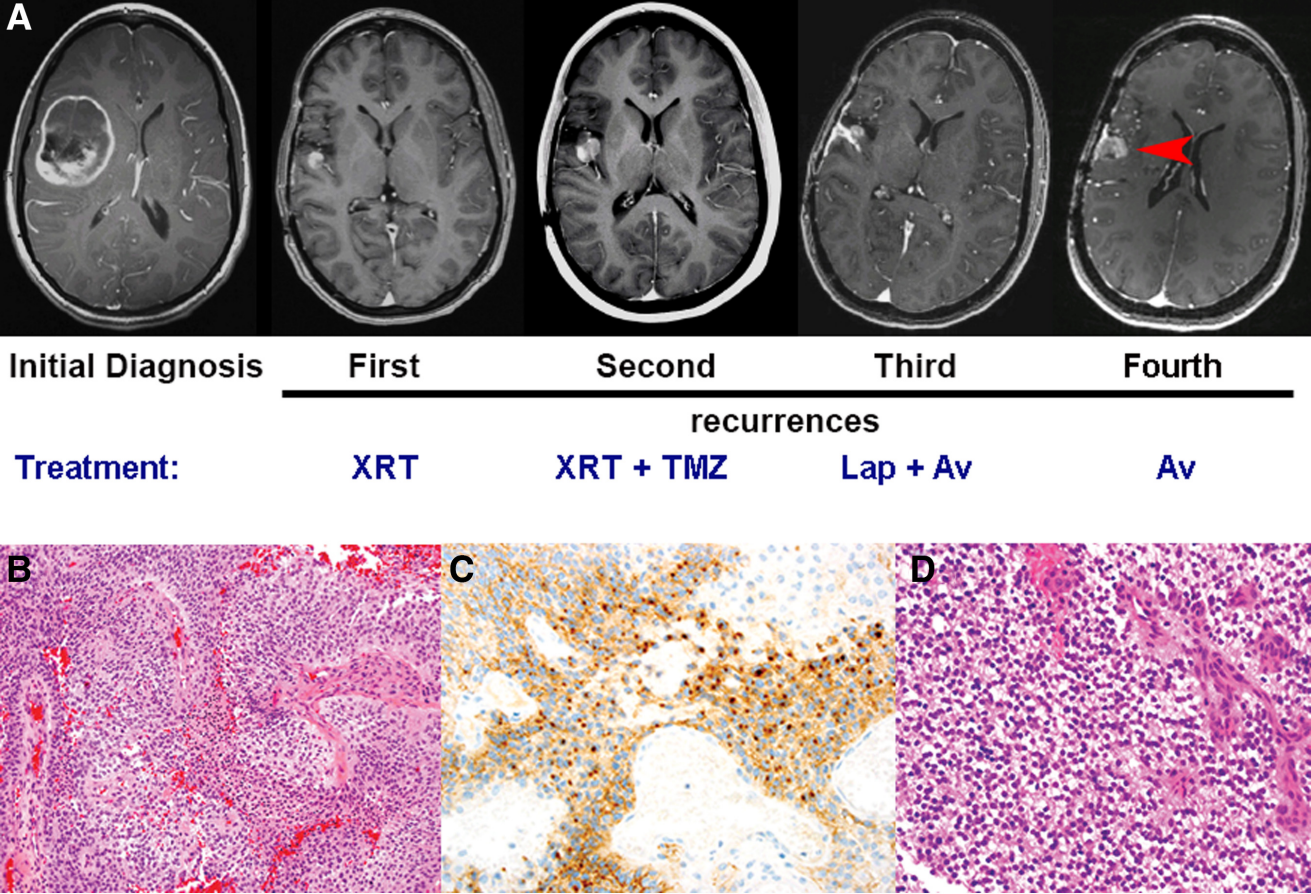
Radiographic and pathological evaluation of initial and recurrent ependymoma. (*A*) Serial MRIs over a 9-yr period demonstrating a heterogeneously enhancing mass in the *right* frontotemporal region at the time of initial diagnosis and four enhancing recurrent lesions adjacent to the initial resection cavity. (*B*) Hematoxylin and eosin (H&E) stain of formalin-fixed paraffin-embedded primary resection material revealed a densely cellular tumor with increased mitotic activity, necrosis, and microvascular proliferation. (*C*) Immunostain for epithelial membrane antigen (EMA) shows multifocal perinuclear dot-like positivity, which is characteristic of ependymal differentiation along with concomitant cytoplasmic expression of glial fibrillary acidic protein (not shown). (*D*) H&E stain of each recurrent tumor revealed persistence of the ependymal phenotype. Pictured is the third recurrence. All the photomicrographs are taken at 40× magnification.

The initial diagnosis was made in a 16-yr-old right-handed female who presented to the St. Louis Children's Hospital Emergency Department with a 3-d history of headache and vomiting (Table 1). Magnetic resonance imaging (MRI) scan revealed a 6 × 4 cm enhancing mass in the right frontotemporal region (Fig. 1A, initial diagnosis). The patient underwent a GTR via a right frontotemporal craniotomy. Pathological evaluation was significant for a hypercellular glial tumor with prominent pseudo-rosettes, increased mitoses, vascular proliferation and necrosis, and perinuclear dot-like expression of epithelial membrane antigen (EMA) (Fig. 1B,C) along with diffuse glial fibrillary acidic protein immunoreactivity. A diagnosis of anaplastic ependymoma (WHO Grade III) was made. Evaluations for CNS dissemination were negative. The patient received 59.4 Gy of fractionated photon irradiation to the tumor bed plus a 1-cm margin, which is standard for supratentorial ependymoma. Forty-four months after the initial diagnosis, the patient suffered a seizure and an MRI revealed a 13 × 15 × 16 mm nodular recurrence in the right frontal lobe along the posterior margin of the initial resection cavity (Fig. 1A, first recurrence). MRI of spine and cerebrospinal fluid cytology were negative. The patient underwent complete resection of the recurrent tumor, which exhibited similar histology to the initial tumor. The resection cavity and margin were reirradiated with an additional 59.4 Gy of fractionated photon irradiation and the patient received 10 mo of standard dose temozolomide treatment.

**Table 1.** Clinical history

Date	Event
Month 1	Initial GTR of anaplastic ependymoma WHO Grade III
Months 2–3	Irradiation of tumor bed to 59.4 Gy
Month 45	GTR of first recurrent anaplastic ependymoma
Months 47–48	Reirradiation with 59.4 Gy with 10 months of temozolomide
Months 48–58	Temozolomide chemotherapy
Month 63	GTR of second recurrent anaplastic ependymoma
Month 65	Lapatinib and Avastin therapy initiated
Month 69	Lapatinib discontinued secondary to toxicity
Month 77	Avastin discontinued
Month 83	GTR of third recurrence of anaplastic ependymoma
Months 84–104	Avastin therapy
Month 104	GTR of fourth recurrent anaplastic ependymoma

GTR, gross total resection; WHO, World Health Organization.

A surveillance scan 17 mo after the second resection demonstrated a 7-mm enhancing nodule in the temporal surface of the right sylvian fissure near the resection cavity, consistent with recurrence (Fig. 1A, second recurrence). Following a third complete resection, histopathology was again consistent with anaplastic ependymoma and analysis for dissemination was negative. The patient was enrolled on CERN-0801 at Children's Memorial Hospital in Chicago and received combined Avastin and lapatinib. Lapatinib was discontinued 4 mo later because of toxicity, and Avastin was continued for an additional 8 mo for a total of 1 yr of treatment every 2 wk. Six months later, an MRI revealed a new right perisylvian lesion and right thalamic enhancing nodule (Fig. 1A, third recurrence). Complete resection of the perisylvian lesion was performed and pathology again indicated anaplastic ependymoma (Fig. 1D) with no evidence of dissemination. Avastin was restarted and continued for 20 mo until new evidence from serial MRI indicated progression in a perisylvian lesion that had remained following the most recent surgery (Fig. 1A, fourth recurrence). This lesion also was completely resected and diagnosed as anaplastic ependymoma. The patient continues on treatment at the time of this report, >11 yr from original diagnosis, without evidence of dissemination beyond this loco-regional area.

## GENOMIC ANALYSES

### Analysis of the Matched Normal Sample

To determine whether the patient possessed a germline predisposition to cancer, we analyzed the sequence data obtained from her leukocyte-derived DNA (normal, Supplemental Table S1) and identified 176 protein-altering constitutional variants that were rare in the population and fell into highly damaging classes of mutations (frameshifts, nonsense, nonstop, or splice-site). Variants were observed in several genes known to be important for immune function, including splice site SNPs in *RAG1*, *HLA-DRB1*, and *HLA-DRB5*, as well as a nonsense mutation in *HLA-DRB5.* Several cancer-relevant genes were also observed: splice-site alterations in *DDX3X* (Dahlin et al. 2015) and *MAD2L2* (alias: *REV7*) (Boersma et al. 2015; Xu et al. 2015) and in-frame insertions in *MNX1* (Das 2016) and *ZFHX3* (Mabuchi et al. 2010)*.* Some with direct glioma relevance were also observed: *FOXD1* (in-frame deletion) (Koga et al. 2014; Cheng et al. 2016; Gao et al. 2017), *BCL2L2* (SNP) (Chung et al. 2015), and *RYK* (frameshift insertion) (Adamo et al. 2017). Although *MNX1* functions as an oncogene to promote pancreatic islet cell tumors in multiple endocrine neoplasia type 1 (MEN1) (Scacheri et al. 2006), this particular mutation is common in the population and unlikely to be relevant to predisposition.

### Landscape of Somatic Mutations during Disease Progression

**Figure 2.**
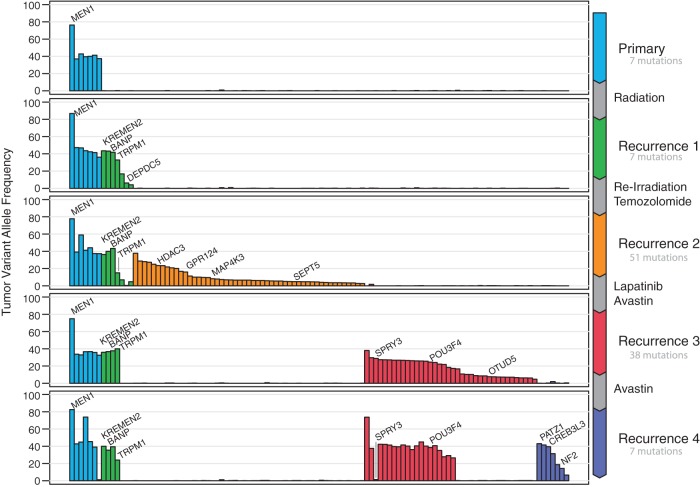
Variant allele fractions of nonsilent mutations in protein-coding genes in all five resections.

We identified 1332 somatic mutations across the five resection specimens, 162 of which were in protein-coding regions, and 110 of which were nonsilent (Fig. 2; Supplemental Table S2). The primary tumor sample contained only one overtly cancer-related gene mutation, an expressed frameshift insertion in *MEN1* (K237fs) (Table 2). We also observed several large copy-number alterations (CNAs) in this sample, including deletions of 6p, 15q, 22, and the first 22 Mb of Chromosome 1, that were shared with the recurrent tumors (Supplemental Fig. S1, Table S3). Chromosome 11 was heavily rearranged, with multiple distinct regions of amplification and deletion, one of which deleted the second copy of *MEN1*. Integrated analysis of the DNA and RNA did not detect any gene fusion events, although many putative structural variants were detected (Supplemental Tables S4 and S5).

**Table 2.** Functions and cancer-relatedness of mutated genes

Category	Gene	Chrom	HGVS DNA reference	HGVS protein reference	Variant type	Predicted effect	dbSNP ID	Genotype	Comments	PMID references
Epigenetic modifiers	*MEN1*	11	ENST00000337652.5:c.711_712insG	ENSP00000337088.1:p.Lys237GlnfsTer?	frame_shift_ins	p.K237fs	–	hom	H3K4 trimethylation, DNA methylation	23850066
	*GON4L*	1	ENST00000368331.1:c.2455A>G	ENSP00000357315.1:p.Asn819Asp	missense	p.N819D	–	het	HDAC1 interactor	21454521
	*HDAC3*	5	ENST00000305264.3:c.50_55+5del	ENSP00000302967:p.His17?	frame_shift_del	p.H17fs	–	het	Histone deacetylase	25313724
	*SETD9*	5	ENST00000285947.2:c.788_796del	ENSP00000285947.2:p.Ile263_Tyr265del	in_frame_del	p.IAY263in_frame_del	–	het	Histone methyltransferase	20930037
	*BANP*	16	ENST00000286122.7:c.668A>G	ENSP00000286122.7:p.Asn223Ser	missense	p.N223S	–	het	Recruits HDAC1 and deacetylation of H3K9	16166625
	*SUV39H1*	X	ENST00000337852.6:c.448C>T	ENSP00000337976.6:p.Arg150Cys	missense	p.R150C	rs368779259	het	H3K9 trimethylase	26807716
	*FAM208A*	3	ENST00000355628.5:c.97G>A	ENSP00000347845.5:p.Glu33Lys	missense	p.E33K	–	het	Component of HUSH complex; required for H3K9me3	26022416
	*POU3F4*	X	ENST00000373200.2:c.1013C>T	ENSP00000362296.2:p.Pro338Leu	missense	p.P338L	–	het	Epigenetics of neuronal fate	23933087
	*PATZ1*	22	ENST00000266269.5:c.562G>T	ENSP00000266269.5:p.Asp188Tyr	missense	p.D188Y	–	het	Regulates chromatin openness and pluripotency	25515777
	KAT6B	10	ENST00000287239.4:c.3827C>T	ENSP00000287239.4:p.Pro1276Leu	missense	p.P1276L	–	het	Histone acetyltransferase	26208904
Intracellular signaling	*KREMEN2*	16	ENST00000303746.5:c.494G>T	ENSP00000304422.5:p.Gly165Val	missense	p.G165V	–	het	Inhibitor of WNT signaling	20846389
	*DEPDC5*	22	ENST00000266091.3:c.2939G>A	ENSP00000266091.3:p.Trp980Ter	nonsense	p.W980*	–	het	Inhibitor of mTORC signaling	23723238
	*NET1*	10	ENST00000355029.4:c.498G>T	ENSP00000347134.4:p.Glu166Asp	missense	p.E166D	–	het	rhoGEF required for proliferation	23864709
	*SPRY3*	X	ENST00000302805.2:c.55C>T	ENSP00000302978.2:p.Arg19Cys	missense	p.R19C	–	het	Inhibitor of FGF signaling	18219583
	*VBP1*	X	ENST00000286428.5:c.331C>A	ENSP00000286428.5:p.Leu111Met	missense	p.L111M	–	het	VHL protein interactor	23964080
	*ARHGAP32*	11	ENST00000310343.9:c.4703C>G	ENSP00000310561.8:p.Thr1568Ser	missense	p.T1568S	–	het	Inhibitor of RHOA, CDC42, and RAC1 signaling	12857875
	*OTUD5*	X	ENST00000156084.4:c.1702C>A	ENSP00000156084.4:p.Pro568Thr	missense	p.P568T	–	het	Activator of p53	24143256
	*KLHL21*	1	ENST00000377658.4:c.501G>C	ENSP00000366886.4:p.Glu167Asp	missense	p.E167D	–	het	Regulator of mitosis	19995937
	*NF2*	22	ENST00000338641.4:c.551G>A	ENSP00000344666.4:p.Trp184Ter	nonsense	p.W184*	–	het	Tumor suppressor, regulator of hippo pathway	25893302
	*LATS1*	6	ENST00000253339.5:c.2365G>C	ENSP00000253339.5:p.Asp789His	missense	p.D789H	–	het	Mediator of hippo pathway, regulated by NF2	25026211
	*MAP4K3*	2	ENST00000263881.3:c.899T>C	ENSP00000263881.3:p.Phe300Ser	missense	p.F300S	–	het	Component of MAPK pathway regulator of LATS1	26437443
	*TRAF3*	14	ENST00000392745.2:c.53C>G	ENSP00000376500.2:p.Pro18Arg	missense	p.P18R	–	het	TNFR-associated regulator of MAPK and NF-κB pathways	28098136
Metabolism	*LETM1*	4	ENST00000302787.2:c.286G>A	ENSP00000305653.2:p.Val96Met	missense	p.V96M	–	het	Mitochondrial structural protein	25077561
	*TXNRD2*	22	ENST00000535882.1:c.1429G>C	ENSP00000439314.1:p.Ala477Pro	missense	p.A477P	–	het	Thioredoxin reductase 2	25647640
	*HSD3B2*	1	ENST00000369416.3:c.1004G>A	ENSP00000358424.3:p.Arg335Gln	missense	p.R335Q	–	het	Required for steroid biosynthesis	22262841
	*GLB1*	3	ENST00000445488.2:c.2047G>C	ENSP00000393377.2:p.Ala683Pro	missense	p.A683P	–	het	β-galactosidase	23011886
	*MT-ND4*	MT	ENST00000361381.2:c.279del	ENSP00000354961.2:p.Lys93AsnfsTer?	frame_shift_del	p.K93fs	–	het	Core component of mitochondrial NADH dehydrogenase	25909222
Neuro-developmental disorders	*SYNE1*	6	ENST00000265368.4:c.11097C>G	ENSP00000265368.4:p.Phe3699Leu	missense	p.F3699L	–	het	SYNE1 ataxia	27086870
	*GPR124*	8	ENST00000412232.2:c.1316_1334del	ENSP00000406367.2:p.Asn439ThrfsTer16	frame_shift_del	p.N439fs	–	het	CNS angiogenesis	21071672
	*CACNG2*	22	ENST00000300105.6:c.541T>C	ENSP00000300105.6:p.Tyr181His	missense	p.Y181H	–	het	Bipolar disorder	25730879
	*SH3TC2*	5	ENST00000515425.1:c.3016del	ENSP00000423660.1:p.Ser1006ProfsTer9	frame_shift_del	p.S1006fs	–	het	Charcot–Marie–Tooth	20028792
Other cancer-related genes	*SLC39A11*	17	ENST00000542342.2:c.616G>T	ENSP00000445829.2:p.Val206Phe	missense	p.V206F	–	het	Ovarian cancer	26091520
	*TRPM1*	15	ENST00000542188.1:c.334A>C	ENSP00000437849.1:p.Ile112Leu	missense	p.I112L	–	het	Melanoma	22897572

hom, homozygous; het, heterozygous; frame_shift_ins, frameshift insertion; frame_shift_del, frameshift deletion.

All SNVs, indels, and CNAs found in the initial resection were retained in the first recurrence, which was diagnosed after radiation therapy and a 44-mo interval. An additional 12 new protein-coding somatic mutations were identified in the recurrent tumor, including a nonsense mutation in *DEPDC5*, an inhibitor of mTORC signaling. Missense mutations were observed in *KREMEN2* (G165V), a gene that has been linked to melanoma, and in *BANP* (N223S), an epigenetic regulator. None is obviously expressed in this tumor, but the variants in both *KREMEN2* and *BANP* are expressed in subsequent tumors with higher quality and higher-depth RNA-seq, so it is likely that these variants are expressed below our level of sensitivity in this resection sample. Mutated *DEPDC5* may have been undetectable because of undergoing nonsense-mediated decay.

The second recurrent tumor emerged after additional radiation therapy and treatment with temozolomide. It was resected and the genomic analysis of this specimen indicated that essentially all previously observed mutations were retained, with the exception of two low-VAF protein-coding variants from the previous recurrence, including loss of the *DEPDC5* nonsense mutation. An additional 66 protein-coding SNVs and indels were acquired, including a 19-bp frameshift deletion in *GPR124* and low-VAF missense mutations in *SEPT5* (T260A), *MAP4K3* (F300S), and *KAT6B* (P1276L). Of these, only the *MAP43K* and *KAT6B* mutations were observably expressed. The copy-number landscape was identical to the previous tumors, with the exception of a new homozygous deletion on Chromosome 2p.

The third recurrence occurred after treatment with Avastin and lapatinib. Genomic analysis of this resection specimen revealed that all coding mutations specific to the second recurrence, including the Chromosome 2 copy-number loss, were undetectable at the third recurrence. In contrast, virtually all mutations identified in the first two resections persisted, the only exception being two low-VAF events in *MYH10* and *OR1L1*. Fifty-six new protein-coding mutations were acquired, including missense mutations in *POU3F4* (P568T), an epigenetic regulator, *OTUD5* (P338L), a p53 activator, and *SPRY3* (R19C), a regulator of FGF signaling. None has been previously implicated in ependymoma, and their relevance for disease progression and therapy resistance is unclear.

The fourth recurrence was resected after continued Avastin treatment. In this sample, 29 of the protein-coding mutations newly acquired in the prior (third) recurrence were no longer detected, but 18 new protein-coding mutations were identified. These included nonsense mutations in *CREB3L3* and *NF2*, a gene previously linked to ependymoma. A missense mutation in the chromatin/transcriptional regulator *PATZ1* was also observed. In addition to the *NF2* mutation, we identified two point mutations that potentially impact Hippo pathway signaling in *LATS1* and *MAP4K3* (Meng et al. 2015; Oh et al. 2015).

### Clonal Heterogeneity and Mechanisms of Tumor Evolution

**Figure 3.**
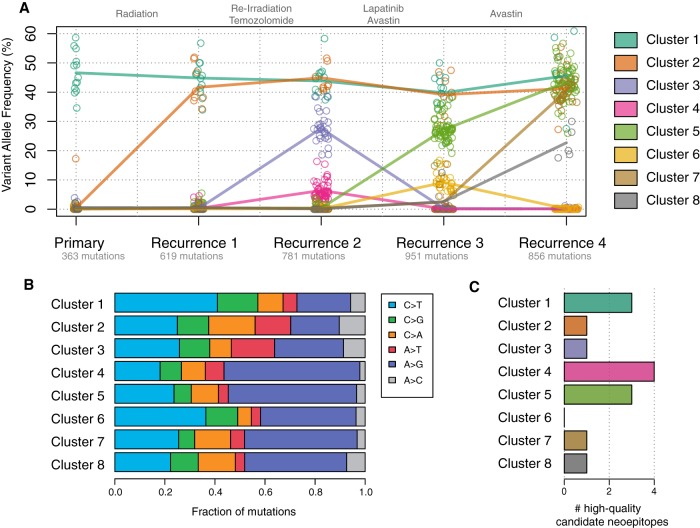
(*A*) Subclonal clustering of the five tumor samples. Points represent the VAFs of individual SNVs at each time point, and lines connect the mean VAF of each cluster in each sample. Each sample is labeled with the number of detectable mutations at that time point. (*B*) Mutation spectrum of each cluster. (*C*) The number of high-quality MHC Class I neoantigens found in each subclonal population.

To characterize the changing clonal architecture of this tumor, the variant allele fractions of copy-number neutral SNVs were clustered in five dimensions using the sciClone algorithm (Fig. 3A). Eight clusters were detected, and the mutation spectrum for each was identified. The first recurrent tumor after radiation therapy was dominated by cluster 2, which emerged from a population of cells undetectable in our analysis of the original biopsy data (with a sensitivity of ∼2% VAF). The mutation spectrum shows a notable decrease in C>T transitions in cluster 2, when compared with those in cluster 1 from the original tumor (Fig. 3B).

In the second recurrence, following additional radiation and treatment with temozolomide, we identified the emergence of two new subclonal populations (clusters 3 and 4) that were likewise undetectable in the prior two samples’ data. Cluster 4, and all subsequently appearing clusters, each have a significantly higher proportion of A>G transitions than the founding clone, a pattern consistent with temozolomide-induced mutagenesis (all *P* < 0.03) (Supplemental Table S6; Bodell et al. 2003). In the third recurrence, following Avastin treatment, both clusters 3 and 4 were undetectable, but clusters 5 and 6 emerged. Although cluster 6 was cleared in the final resection sample we studied, cluster 5 persisted and two rare subclonal populations expanded into clusters 7 and 8, which make up a substantial portion of the final tumor. Some mutations in these two clusters were just above the level of detection in the third resection.

Though there were too few mutations to perform per-cluster mutational signature analysis (Rosenthal et al. 2016), we did compare the deletion/substitution ratio between mutations that predated irradiation (cluster 1) and those that arose after radiotherapy (clusters 2–8). We observed a significant increase in the proportion of deletions postirradiation (Pearson's χ^2^ *P* = 1.657 × 10^−05^), a finding that is consistent with previously reported mutational signatures of ionizing radiation (Behjati et al. 2016).

In addition to identifying mutations correlated with specific subclonal expansions, we also examined the expression of *O*^6^-methylguanine DNA methyltransferase (*MGMT*), which is known to drive brain tumor recurrence through increased expression in post-temozolomide lesions (Bocangel et al. 2002; Hegi et al. 2005). In this case, *MGMT* RNA expression levels were not clearly correlated with the emergence of post-temozolomide recurrences, suggesting that they relied upon alternative mechanisms of resistance (Supplemental Fig. S2).

### Evolving Landscape of Targets for Immunotherapy

To understand how the immunogenicity of this tumor evolved over the course of treatment, we applied the pVACSeq neoantigen prediction pipeline (Hundal et al. 2016) to the protein-altering mutations that we observed in each tumor studied. The patient's HLA haplotypes were inferred to be A*24:02, A*26:01, B*40:02, B*38:01, C*12:03, and C*03:05. We identified only 14 expressed mutations that produce “high-quality” predicted MHC Class I neoantigens (Fig. 2C), which we define as having median binding affinity (ic50) of <500 nM, and with a higher binding affinity to the mutant than the wild-type peptide (Supplemental Table S7). As overall mutation burden is highly correlated with neoantigen load, this is perhaps unsurprising. Only three neoantigens were present in the founding clone, whereas 11 of the 14 were specific to a subclonal population and therefore not present in all cells of the tumor.

## DISCUSSION

In this study, we found that standard-of-care and experimental approaches to treatment of an ependymoma increased its mutational burden and diversified its subclonal architecture. The diagnosis of a supratentorial anaplastic ependymoma, not arising from the ventricles, is uncommon. Ependymomas, which occur in both pediatric and adult patients, display age-dependent patterns of location and histology. Overall, the majority of ependymomas occur in the spine, though this location is involved in only 20% of pediatric cases (McGuire et al. 2009a, 2009b). As was evident in this case, supratentorial ependymomas are most common in older children and adolescents. In addition, anaplastic histology is also more common in pediatric cases. Recent genomic analyses have indicated that most supratentorial anaplastic ependymomas are associated with fusion events involving RELA or YAP1 (Pajtler et al. 2015). Neither of these fusion events was detected in this case.

### The Role of *MEN1* Mutations

Notable in the evaluation of the primary diagnostic specimen was the biallelic somatic disruption of *MEN1*. *MEN1* mutations, typically biallelic, have been infrequently reported in ependymoma, both in the context of familial MEN1 syndrome and sporadically (Kato et al. 1996; Urioste et al. 2002; Al-Salameh et al. 2010; Lee et al. 2016b), and appear to occur in both high- and low-grade tumors of any location. Although MEN1 mutation has been associated with recurrence and progression from grade II to III ependymoma (Urioste et al. 2002; Funayama et al. 2013), we offer the first clear evidence that MEN1 mutation is a driver of the founding clone.

Functionally, *MEN1* mutations are known to increase *DNMT1* activity, which leads to global increases in CpG island methylation, a characteristic of other tumors (Funayama et al. 2013; Mack et al. 2014; Yuan et al. 2016), and silences key tumor suppressor genes, including *CDKN1B, CDKN2A, APC*, and *RASSF1A* (Karnik et al. 2005; Lindberg et al. 2008; Juhlin et al. 2010). Coupled with the observed mutations in other epigenetic regulators, these data add new evidence for epigenetic dysregulation in the genesis and progression of ependymoma.

Loss of menin function also leads to activation of the RAS (Wu et al. 2012), MAP kinase (Gallo et al. 2002), PI3 kinase (Wang et al. 2011), Sonic Hedgehog (Gurung et al. 2013), Wnt (Cao et al. 2009), and TGF-β signaling pathways, all with established roles in gliomagenesis (Matkar et al. 2013). Their role is underscored by the accumulation of mutations in additional regulators of their activation during the course of this tumor's treatment: MAPK (*SPRY3*) (Cabrita and Christofori 2008), PI3K (DEPDC5) (Cabrita and Christofori 2008; Bar-Peled et al. 2013), and WNT (*KREMEN2*, *NET1*, *GPR124*) (Orlow et al. 1987; Mao et al. 2002; Posokhova et al. 2015; Wei et al. 2017).

### Drivers of Recurrence and Susceptibility

Although more cases will be needed to confidently identify the specific mutations that drive the expansion of therapy resistant subclones, the Hippo pathway stands out as a compelling potential driver of the fourth recurrence, induced by new mutations in *LATS1* (missense) and in *NF2* (nonsense). *NF2* loss-of-function mutations have been previously associated with spinal ependymomas (Lee et al. 2016a), and recent evidence suggests that *YAP1*, the nuclear target of Hippo signaling, mediates aberrant proliferation upon *NF2* loss during tumorigenesis (Shi et al. 2016). Furthermore, oncogenic *YAP1* activation occurs as a consequence of a loss in *NF2*-dependent inactivation of *LATS1* (a key inhibitor of *YAP1*), and decreased *LATS1* activity has also been associated with glioma progression (Ji et al. 2012; Oh et al. 2015; Shi et al. 2016). YAP1 fusions are a key characteristic of one subgroup of supratentorial ependymomas (Archer and Pomeroy 2015) and we hypothesize that these mutations may represent convergent evolution, producing similar phenotypic effects via a different mechanism.

Only one substantial subclone was clearly responsive to therapy (cluster 3) and it was eradicated after treatment of recurrence 2 with Avastin and lapatinib. Among the compelling target mediators of response or biomarkers of response is the mutation in *GPR124*. This orphan member of the adhesion G protein–coupled receptor family is required specifically for the development of the brain vasculature in a VEGF-dependent manner (Kuhnert et al. 2010; Cullen et al. 2011; Zhou and Nathans 2014), and GPR124 may be a biomarker of Avastin response (Wang et al. 2014). GPR124 activates canonical Wnt signaling, which, as described above, is normally directly inhibited by MEN1 and KREMEN2. The genes for both of these proteins were mutated in this tumor, suggesting a model for Avastin response that might involve enhanced activation of a VEGF–Wnt axis.

### Changes to the Neoantigen Landscape during Progression

Overall, the number of expressed MHC Class I neoantigens that we predicted was low, as expected in a tumor with only 110 protein-altering mutations. The presence of a relatively high burden in the founding clone suggests that mechanisms of immune evasion were already present when the initial tumor presented and may explain why there was no relationship between neoantigen load and subclonal response in subsequent tumors. This is supported by the observation that expression markers of T-cell activation were low in all five tumors.

### Origin of New Subclones in Later Recurrences

Although we present evidence suggesting that radiation and temozolomide treatment increased the mutation burden of this tumor, new mutations continued to be observed after their use was discontinued. It is important to note that this is not necessarily indicative of a continued elevated mutation rate. Mutations first observed in the last two recurrences may have existed at very low frequencies in prior time points, and the observed mutation spectrum in the new subclones is consistent with damage from previous therapies. Our prior work with ultradeep sequencing suggests that many rare subclones often exist in a tumor (with neutral fitness) and only become detectable after their fitness increases, either via acquisition of one or more driver mutations or because the therapeutic regimen changes the environment (Griffith et al. 2015b; Uy et al. 2017).

### Future Directions

These results suggest that radiation and chemotherapy contributed to the increasing complexity of this tumor by both adding to the mutational burden and expanding the subclonal architecture. Determining whether this natural history is generally true in ependymoma progression, and what impact therapy-induced tumor evolution has on outcome, is an important area of investigation with the potential to alter how we treat patients with both completely resected supratentorial ependymoma and other brain tumors that are treated with irradiation but frequently recur. In the largest published study of ependymoma outcome involving 282 patients, GTR was the only prognostic factor associated with increased survival (Vera-Bolanos et al. 2015). In that analysis, GTR and postsurgical radiation therapy were associated with a shorter progression-free survival than GTR alone. Data presented here raise the alarming hypothesis that time-to-progression was shortened because irradiation promoted tumor evolution.

These clinical observations together with the sequencing-based characterizations presented here suggest that under some circumstances, adjuvant therapy may not be providing a benefit, and indeed may hasten recurrence by promoting molecular diversification of the tumor. We propose that this phenomenon be studied prospectively. In particular, our data suggest that completely resected supratentorial ependymomas, and possibly other brain tumors, should be sequenced at the time of diagnosis and again if there is a recurrent tumor. Over time, this might reveal the genotypes for which radiation therapy eradicates the founding clone, resulting in a cure, and in those for which it does not, but instead contributes to evolving tumor complexity. Ultimately, it may be prudent to initially observe those patients with complete resections without additional therapy or to treat those patients whose tumors are likely to evolve in response to radiation therapy with targeted agents only as dictated by genomic analysis. Critically important to this effort will be the use of unbiased sequencing approaches like whole-exome or whole-genome sequencing rather than sequencing of targeted gene panels. Although identification of druggable targets is important, it may be equally important to construct more global models of tumor evolution.

Finally, it will be important to investigate further the utility of genomic characterization to inform therapeutic options in this disease type. Although not all of the variants we identified were “druggable” in the classical sense, a subset were found to comprise predicted high-affinity neoantigen targets that, ultimately, formed the basis of a polyvalent personalized vaccine, administered after recurrence 4. Such cancer immunogenomics approaches to clinical care are only made possible through comprehensive genomic approaches to tumor characterization. Although the efficacy of these treatments awaits large-scale studies and clinical trials that are ongoing, our case highlights the potential to consider the pursuit of a personalized vaccine in extremely challenging settings of multiply recurrent disease such as the one herein, where few to no other options exist.

## METHODS

### DNA Sequencing

DNA was isolated from fresh frozen sections of each tumor resection using the QIAGEN Dual Prep and evaluated for quality and concentration using established methods. DNA was isolated from PBMC after Ficoll-based isolation to provide a normal comparator and was evaluated for quality and concentration. Using 500 ng input for all five tumors and the blood normal DNA, we generated two indexed whole-genome sequencing libraries by standard methods (Kapa Biosystems) for each sample. One library per sample was processed through exome hybrid capture using the IDT xGEN research exome capture reagent (Integrated DNA Technologies), quantitated and amplified postcapture using the manufacturer's protocol. Each of the corresponding WGS libraries was amplified by PCR, quantitated, and diluted as appropriate for Illumina sequencing. The final libraries for each sample (WGS + exome) were pooled to produce combined tumor and normal WGS- and exome-sequencing data in a specific proportion, yielding ∼10-fold WGS and ∼1000-fold exome coverage (Supplemental Table S1). The resulting library pools were loaded onto the HiSeqX platform and sequenced using 150-bp paired end reads.

### Somatic Variant Analysis

After index-based binning of the reads into WGS- and exome-derived tumor and normal data, sequence data were aligned to reference sequence build GRCh37-lite-build37 using BWA-mem (Li, H. arXiv:1303.3997 [q-bio.GN]) version 0.7.10 (params: -t 8::), then merged and deduplicated using Picard version 1.113 (https://broadinstitute.github.io/picard/). Somatic variants were called from the combined data using our Genome Modeling System (Griffith et al. 2015a) as follows.

SNVs were detected using the union of four callers: (1) SAMtools (Li et al. 2009) version r982 (params: mpileup -BuDs) intersected with Somatic Sniper (Larson et al. 2012) version 1.0.4 (params: -F vcf –G -L -q 1 -Q 15) and processed through false-positive filter v1 (params: --bam-readcount- version 0.4 --bam-readcount-min-base-quality 15 --min-mapping-quality 40 --min-somatic-score 40), (2) VarScan (Koboldt et al. 2012) version 2.3.6 filtered by varscan-high-confidence filter version v1 and processed through false-positive filter v1 (params: --bam-readcount-version 0.4 --bam-readcount-min-base-quality 15), (3) Strelka (Saunders et al. 2012) version 1.0.11 (params: isSkipDepthFilters = 0), and (4) Mutect (Cibulskis et al. 2013) v1.1.4.

Indels were detected using the union of three callers: (1) GATK (McKenna et al. 2010) somatic-indel version 5336; (2) VarScan version 2.3.6 filtered by varscan-high-confidence-indel version v1, and (3) Strelka version 1.0.11 (params: isSkipDepthFilters = 0).

SNVs and Indels were further filtered by removing artifacts found in a panel of 905 normal exomes, removing sites that exceeded 0.1% frequency in the 1000 genomes or NHLBI exome-sequencing projects, and then using a Bayesian classifier (https://github.com/genome/genome/blob/master/lib/perl/Genome/Model/Tools/Validation/IdentifyOutliers.pm) and retaining variants classified as somatic with a binomial log-likelihood of at least 10.

For protein-coding mutation counts described in the results below, a variant was considered to be present in a sample if it appeared with at least three variant supporting reads and a VAF of >2.5%. As some sites had low or variable coverage, a variant was only considered to be completely cleared if it did not appear in any subsequent samples.

Copy-number aberrations were detected using bam-window (window-size 10,000) and copy-cat version 1.6.11 (params: --per-read-length –per-library) (https://github.com/chrisamiller/copyCat). Uneven sequence coverage of the normal sample required us to run copyCat in tumor-only mode, followed by manual review to differentiate somatic from germline copy-number events.

Putative structural variants were detected using the union of BreakDancer 1.4.5 (Chen et al. 2009) filtered by novo-realign and tigra-sv, and squaredancer 0.1 (https://github.com/genome/genome/blob/master/lib/perl/Genome/Model/Tools/Sv/SquareDancer.pl).

### RNA Sequencing

Total RNA was concurrently isolated from each fresh frozen tumor resection (QIAGEN Dual Prep), and evaluated for quality and concentration using the Agilent Tapestation. RNA-seq libraries were constructed using the TruSeq Stranded RNAseq library kit (Illumina, Inc.) according to the manufacturer's protocol, quantitated and diluted for sequencing. Using the HiSeq 2500, we produced sequencing data from each RNA-seq library in a single flow cell lane by paired end 100 bp reads, yielding between 96 and 655 million reads per sample. The fourth recurrence sample was subjected to a capture step before sequencing, using the IDT xGEN research exome capture reagent. This sample yielded 856 million reads, with a much higher coding-region percentage (Supplemental Table S1).

### RNA-seq Analysis

The resulting read data were aligned to the human reference with TopHat v2.0.8 (denovo mode, params: --library-type fr-firststrand --bowtie-version=2.1.0). Expression levels were calculated with Cufflinks v2.1.1 (params: --max-bundle-length 10000000 --max-bundle-frags 10000000) (Trapnell et al. 2012).

### Gene Fusions

Gene fusions were detected from RNA and DNA using Integrate v0.2.0 (Zhang et al. 2015) with default parameters.

### Heterogeneity Analysis

Using the high depth of coverage from combining exome and WGS data sets for these tumors, we characterized the heterogeneity of each tumor specimen and compared it to the others in the series. Here, copy-number-neutral variants and their attendant VAFs were clustered in five dimensions using the sciClone algorithm v1.1 (Miller et al. 2014) (parameters: minimumDepth = 300, maximumClusters=15), followed by phylogeny reconstruction with clonEvol (Dang et al. 2017).

### Neoantigen Predictions

Somatic mutations and RNA-seq data from tumors were input into our pVACSeq pipeline (Hundal et al. 2016). WGS data from the normal blood was used to identify the patient's HLA haplotypes for Class I, using Laminar (Warren et al. 2012). MHC Class I binding predictions were generated through pVACSeq using NetMHC v3.4, as well as five other algorithms from the Immune Epitope Database and Analysis resource (IEDB, iedb.org): netMHC, netmhccons, netmhcpan, pickpocket, smm, and smmpmbec. Predictions were retained if the median score had an ic50 < 500 and better binding of the mutant peptide than the wild type (fold-change > 1). Results were then filtered to require expression of the mutant allele (FPKM > 1 and at least one variant-supporting read in the RNA). These combined data sets were used to identify neoantigenic peptide sequences in all five tumor samples, as illustrated in Figure 3C.

### Pathology Methods

All the resection specimens (original and recurrences) were handled as regular surgical neuropathology cases. Although H&E stain and Ki-67 immunostain were performed on all the specimens, glial fibrillary acidic protein and EMA were limited to the initial and 2014 resections.

## ADDITIONAL INFORMATION

### Data Deposition and Access

Sequence data are available at dbGaP, under accession id phs001461.v1.p1. The Germline mutation list is accessible via the same dbGaP study. The variant was deposited in ClinVar (http://www.ncbi.nlm.nih.gov/clinvar/) and can be found under accession numbers SCV000678435–SCV000678467.

### Ethics Statement

The patient had written and parental consent and the study was approved under Washington University in St. Louis IRB ID no. 201102299.

### Acknowledgments

We thank early analysis efforts on this project from Charles Lu. We also gratefully acknowledge the sample intake, project tracking, and data production teams at the McDonnell Genome Institute. Funding for this project was generously provided from the McDonnell Genome Institute endowment and from the Robert E. and Louise F. Dunn Distinguished Professorship endowment to Washington University School of Medicine. We gratefully acknowledge the patient and her family.

### Author Contributions

C.A.M., R.S.F., J.B.R., and E.R.M. contributed to conceptualization; C.A.M. and T.L. did formal analysis/data curation; S.D., M.D.S., J.B.R., and G.P.D. contributed to resources; C.A.M., J.B.R., and E.R.M. wrote the original draft; C.A.M., S.D., T.L., R.S.F., M.D.S., G.P.D., J.B.R., and E.R.M. did review and editing; C.A.M. did visualization; C.A.M., J.B.R., and E.R.M. supervised the study; and E.R.M. acquired funding.

### Competing Interest Statement

The authors have declared no competing interest.

## Supplementary Material

Supplemental Material
