# Supplementary material for: Resistance-promoting effects of ependymoma treatment revealed through genomic analysis of multiple recurrences in a single patient
Source: Cold Spring Harb Mol Case Stud. 2018 Apr;4(2):a002444. doi: 10.1101/mcs.a002444 (PMC5880262; doi:10.1101/mcs.a002444)
Supplement: Supplemental Material [file supp_4_2_a002444__index.html]

Resistance-promoting effects of ependymoma treatment revealed through genomic analysis of multiple recurrences in a single patient — Supplemental Material 

# Resistance-promoting effects of ependymoma treatment revealed through genomic analysis of multiple recurrences in a single patient

## Supplemental Material

- Supplemental\_Figure\_S1.pdf
- Supplemental\_Figure\_S2.pdf
- Supplemental\_Legends.docx
- Supplemental\_Table\_S1.xls
- Supplemental\_Table\_S2.tsv
- Supplemental\_Table\_S3.xls
- Supplemental\_Table\_S4.xls
- Supplemental\_Table\_S5.xls
- Supplemental\_Table\_S6.xls
- Supplemental\_Table\_S7.xls
