# Supplementary figures and images for: Resistance-promoting effects of ependymoma treatment revealed through genomic analysis of multiple recurrences in a single patient
Source: Cold Spring Harb Mol Case Stud. 2018 Apr;4(2):a002444. doi: 10.1101/mcs.a002444 (PMC5880262; doi:10.1101/mcs.a002444)

■ CN Gain  
■ CN Loss

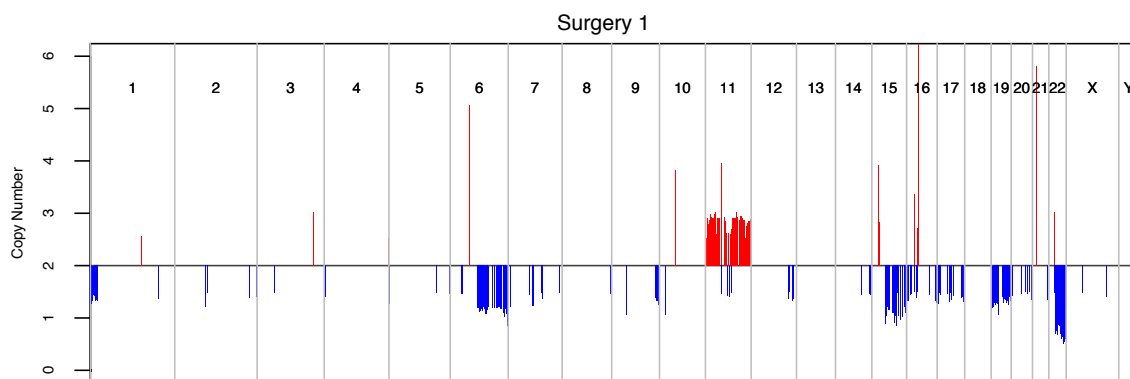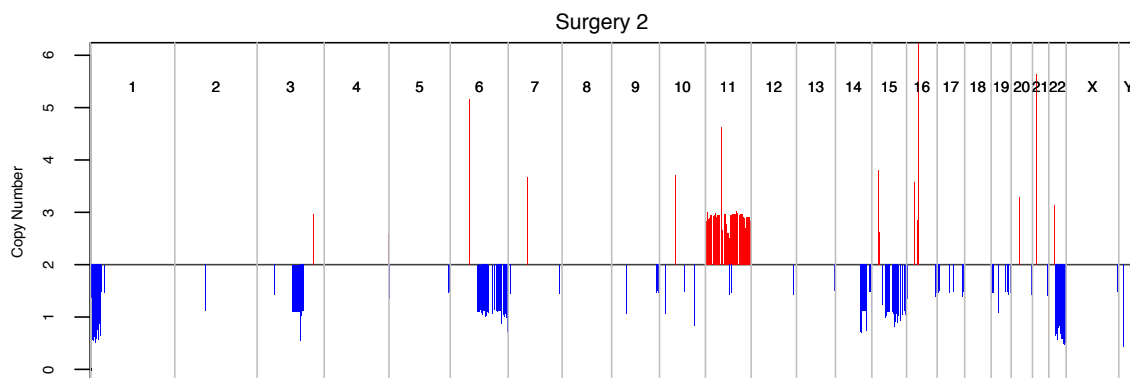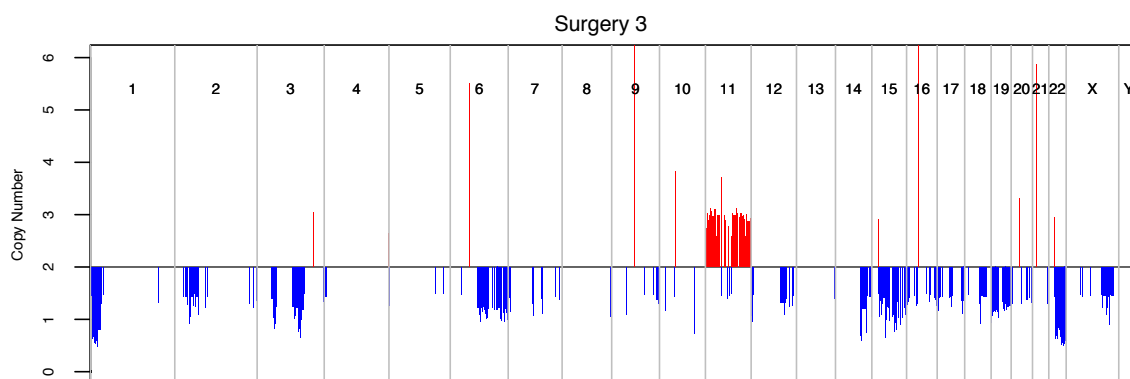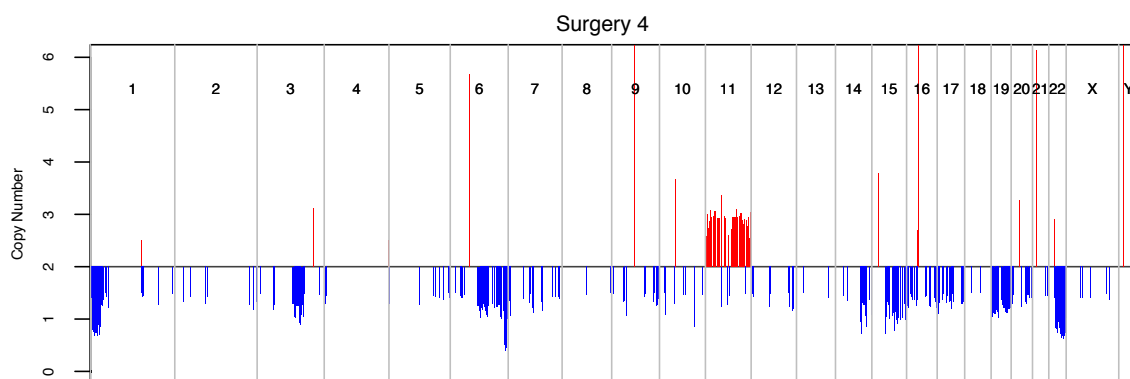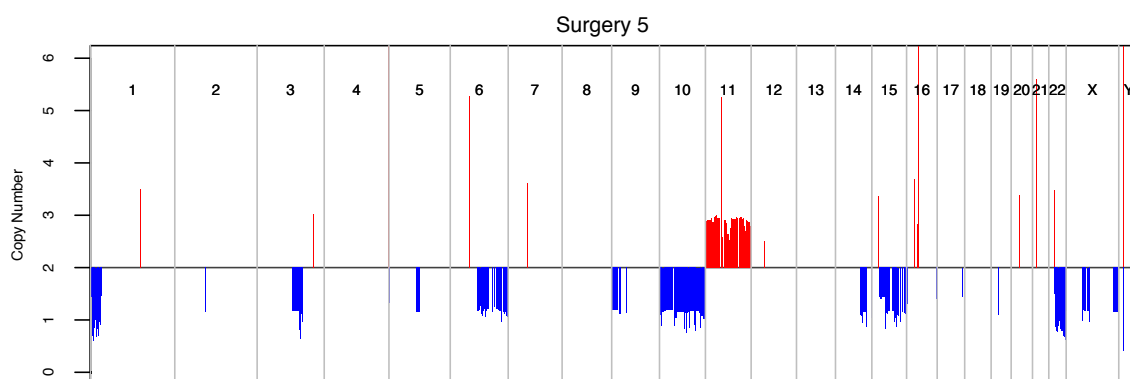

# Surgery 1

Supplement: Supplemental Material [file supp_mcs.a002444_Supplemental_Figure_S1.pdf]

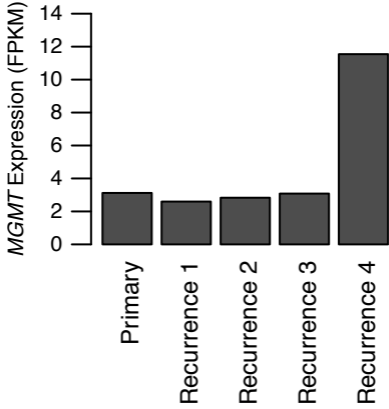

Supplement: Supplemental Material [file supp_mcs.a002444_Supplemental_Figure_S2.pdf]
