# Supplementary material for: Resistance-promoting effects of ependymoma treatment revealed through genomic analysis of multiple recurrences in a single patient
Source: Cold Spring Harb Mol Case Stud. 2018 Apr;4(2):a002444. doi: 10.1101/mcs.a002444 (PMC5880262; doi:10.1101/mcs.a002444)
Supplement: Supplemental Material [file supp_mcs.a002444_Supplemental_Legends.docx]

**Supporting Information Captions**

**S1 Figure:** Copy number alterations found in all 5 samples.

**S2 Figure:** *MGMT* expression from RNA sequencing (units are FPKM). The 4th recurrence sample is presented for completeness, but was sequenced after exome capture, (see S1 Table), which prevents meaningful comparisons to the other samples.

**S1 Table:** DNA and RNA sequencing metrics

**S2 Table:** Somatic variants and readcounts from both DNA and RNA in all resections.

**S3 Table:** Copy number alterations and regions of loss-of-heterozygosity identified in all 5 resections.

**S4 Table:** Gene fusion events identified from RNA sequencing.

**S5 Table:** Structural variants identified in all 5 resections

**S6 Table:** Fraction of A>G transitions in each subclone. P-value calculated by comparing the proportion of A>G mutations in each cluster to those in the founding clone (Cluster 1) using Fisher’s exact test. Multiple-testing correction was applied using the Benjamini-Hochberg method as implemented in the p.adjust() function in R.

**S7 Table:** Predicted high-quality neoantigens identified in all 5 resections.
